# Identification of key genes and diagnostic biomarkers for peripheral atherosclerosis: A multi-omics approach

**DOI:** 10.1097/MD.0000000000042437

**Published:** 2025-05-23

**Authors:** Luofei Huang, Han Li, Quanzhi Lin

**Affiliations:** a Liuzhou Municipal Liutie Central Hospital, Liuzhou, Guangxi, China; b Department of Internal Medicine, Liuzhou People’s Hospital, Liuzhou, Guangxi, China; c Department of Internal Medicine, The First Affiliated Hospital of Guangxi University of Science and Technology, Liuzhou, Guangxi, China.

**Keywords:** diagnostic biomarkers, eQTL, Mendelian randomization, peripheral atherosclerosis, transcriptomics

## Abstract

Peripheral atherosclerosis (PAS), characterized by lipid plaque accumulation in arterial walls, significantly increases cardiovascular risk. This study aimed to identify molecular biomarkers and elucidate underlying mechanisms of PAS progression. We analyzed 2 gene expression omnibus datasets (GSE28829, GSE100927) to identify differentially expressed genes (*P* < .05, |log_2_FC| ≥ 0.585). Functional enrichment (Gene Ontology/Kyoto Encyclopedia of Genes and Genomes) and Mendelian randomization analyses were performed using genome-wide association study and expression quantitative trait loci data. Six hub genes were validated through single-cell RNA sequencing and independent datasets. A diagnostic nomogram was developed and evaluated using calibration curves, decision curve analysis, and receiver operating characteristic metrics. Integrated analysis revealed 6 key PAS-associated genes (leukocyte immunoglobulin-like receptor B1, hematopoietic cell-specific lyn substrate 1, plasminogen activator urokinase, C-type lectin domain family 2 member B, phosphatidylinositol-4-phosphate 5-kinase type 1 beta, cofilin 2). The diagnostic model demonstrated exceptional accuracy, achieving area under the receiver operating characteristic curves of 1.0 (training) and 0.975 (validation). Mendelian randomization confirmed causal relationships, with cofilin 2 and phosphatidylinositol-4-phosphate 5-kinase type 1 beta showing protective effects (odds ratio 0.74–0.90), while C-type lectin domain family 2 member B, hematopoietic cell-specific lyn substrate 1, leukocyte immunoglobulin-like receptor B1, and plasminogen activator urokinase emerged as risk factors. This multi-omics study identifies novel molecular signatures of PAS and establishes a robust diagnostic tool. The findings advance our understanding of PAS pathogenesis and pave the way for personalized therapeutic strategies.

## 1. Introduction

Peripheral atherosclerosis (PAS), a progressive disease characterized by the accumulation of lipids, calcium, and fibrous elements in the peripheral arteries, significantly contributes to morbidity and mortality worldwide.^[1-3]^ The pathogenesis of this condition involves complex interactions between genetic predispositions, environmental factors, and lifestyle choices. Risk factors such as hyperlipidemia, hypertension, smoking, and diabetes play crucial roles in the diseases onset and progression.^[4]^ Despite advancements in pharmacological treatments and interventional strategies for PAS, there are still significant limitations. Current medications, such as statins for lipid lowering and antiplatelet drugs, can only partially control the disease progression and may have side effects. Moreover, these treatments are often based on a one-size-fits-all approach, lacking the ability to be tailored to individual patients’ genetic profiles. There is also a lack of effective biomarkers for early stage diagnosis, which makes it difficult to initiate timely and targeted treatment. Therefore, there is an urgent need to identify new therapeutic targets and develop personalized treatment strategies for PAS.^[5-7]^

Expression quantitative trait loci (eQTLs) are genomic loci that explain variations in gene expression levels within populations and are crucial in elucidating the genetic basis of disease.^[8,9]^ In PAS, eQTL studies have revealed that genetic variants within or near regulatory DNA regions can influence the expression of genes involved in arterial plaque formation and progression.^[10]^ There are 2 main types of eQTLs: cis-eQTLs, which are located near the genes they regulate (usually on the same chromosome within a relatively short distance), and trans-eQTLs, which act on genes located on different chromosomes. In PAS, cis-eQTLs may directly influence the expression of nearby genes involved in arterial plaque formation, such as genes related to lipid metabolism or cell–cell adhesion. Trans-eQTLs, on the other hand, might regulate genes at a distance, potentially coordinating complex biological processes (BPs) across different cell types or tissues involved in PAS pathogenesis. For example, trans-eQTLs could modulate the expression of immune-related genes in monocytes, which play a key role in the inflammatory response associated with PAS.

In addition, Mendelian randomization (MR) analysis provides a powerful way to assess causality in observational studies by using genetic variation as an instrumental variable.^[11,12]^ Unlike traditional observational studies, MR Uses a random classification of gene alleles during gamete formation, similar to Mendels laws of inheritance, to infer a causal relationship between modifiable exposure and disease outcomes.^[13,14]^ By using genetic variation as a proxy for modifiable risk factors, MR Analysis can avoid the confusion and reverse causal bias inherent in traditional epidemiological studies and provide strong evidence for causal inference. In this study, we utilized 2 gene expression datasets from the gene expression omnibus (GEO) database, GSE100927 and GSE28829. Additionally, we employed publicly available eQTL data from the genotype-tissue expression portal and summary data from genome-wide association studies (GWASs) of European ancestry disease cohorts, encompassing 168,832 participants of European descent, to conduct MR analysis. The findings of this study have the potential to revolutionize the management of PAS. By identifying key genes and biomarkers, we can develop novel diagnostic tools with higher sensitivity and specificity for earlystage detection of PAS. Moreover, these discoveries can serve as the basis for personalized treatment strategies, where drugs can be tailored to target specific genetic abnormalities in individual patients. Additionally, the development of a diagnostic nomogram model can improve the prediction of PAS risk, enabling more effective preventive measures and timely interventions, ultimately reducing the morbidity and mortality associated with this debilitating vascular disease.

## 2. Methodology

### 2.1. Collection and processing of GEO data

Our research utilized 2 gene expression datasets from the GEO database (https://www.ncbi.nlm.nih.gov/geo/),^[15]^namely GSE100927 and GSE28829. Dataset GSE28829 was used as the training set. It consists of gene expression arrays with 13 samples of early stage atherosclerotic plaques and 16 samples of advanced atherosclerotic plaques extracted from human carotid arteries. These data in GSE28829 were subjected to background calibration in R, typically by averaging multiple probes for a single gene to determine its expression levels. On the other hand, dataset GSE100927 served as the validation set, which contains 69 samples of non-atherosclerotic and 35 samples of atherosclerotic lesions from human carotid, femoral, and lower popliteal arteries. The selection of GSE100927 and GSE28829 is highly relevant for this study. Both datasets analyze human carotid artery disease, ensuring clinical comparability. GSE28829 focuses on staged atherosclerotic plaques, while GSE100927 includes multi-artery samples (diseased and healthy), broadening tissue representation. Importantly, both use microarray technology, minimizing technical bias and enabling robust integrated analysis. Identification of differentially expressed genes (DEGs) from datasets GSE100927 and GSE28829 was performed using the Limma package in R. The significance threshold was set at a *P*-value < .05 with an absolute log_2_ fold change (FC) ≥ 0.585. To ensure the reliability of detected gene expression changes and minimize potential artifacts from low-quality or spurious data, only genes with a minimum read count of 10 were considered for DEG analysis. For visualization of the DEGs, volcano plots and heatmaps were generated using the “ggplot2” and “heatmap” packages in R, respectively. The gene expression matrices utilized in this study were obtained from publicly available databases; therefore, no additional ethical approval was required.

### 2.2. Analysis of DEGs enrichment utilizing Gene Ontology (GO) annotations and the Kyoto Encyclopedia of Genes and Genomes (KEGG)

The “enrichGO” and “enrichKEGG” packages in R were utilized for DEGs enrichment analysis through GO functional annotations and KEGG pathway enrichment as previous studies.^[16,17]^ An enrichment threshold of *P* < .05 was considered statistically significant. The enriched terms were categorized into BP, cellular component (CC), and molecular function (MF) ontologies, enabling an understanding of the biological roles played by the DEGs.

### 2.3. Design of the MR study

We employed 2-sample MR analysis to evaluate the causal association between gene eQTLs and PAS as previous studies.^[18,19]^ To address the 3 core assumptions of MR causal inference: for the strong association between genetic variants and the exposure, we selected instrumental variables with a significance threshold of 5 × 10^−8^ for their association with eQTLs levels for each gene. This strict threshold ensured a robust relationship between the genetic variants and gene expression levels^[20]^; to minimize the influence of potential confounders on genetic variations and exposure, we used the linkage disequilibrium reference panel from the 1000 Genomes Project. By selecting variants with an *R*^2^ < 0.001 at a distance of 10,000 kb, we reduced the likelihood of confounding due to genetic linkage^[21]^; Regarding the assumption that the exposure is the sole pathway through which genetic variations affect the outcome, we conducted pleiotropy testing using the MR Egger intercept test.^[22]^ If the intercept of the MR Egger regression was not significantly different from 0, it provided evidence that there was no significant horizontal pleiotropy, suggesting that the exposure was likely the main pathway through which genetic variations influenced PAS.

### 2.4. Sources of exposure and outcome data

In this study, eQTLs were employed as the exposure data. eQTLs represent a prevalent type of genetic variation utilized for exploring the association between genotype and gene expression levels. The investigation capitalized on publicly available eQTL data sourced from the genotype-tissue expression portal (https://www.gtexportal.org/home). This dataset encomPASses a diverse array of human tissues and cell types, including but not limited to the heart, liver, and brain. Leveraging this dataset facilitated the assessment of causal relationships between genotypes and particular phenotypes within MR analyses. Additionally, summary data from GWAS were utilized as outcome measures to assess the association with PAS. These GWAS summary data originate from European ancestry disease cohorts, encomPASsing a total of 168,832 participants of European descent (with N_cases_ = 6631 and N_controls_ = 162,201).

### 2.5. Validation of target genes and prediction of pharmacotherapy

We utilized the “VennDiagram” package to identify potential target genes by intersecting the degree data from the experimental group GSE28829 with the geneQTLs dataset after standard filtering. Subsequently, GO and KEGG enrichment analyses were conducted to comprehensively elucidate the biological functions and underlying mechanisms of these target genes. Next, single-cell transcriptomic data (GSE159677) were obtained from the GEO database. The dataset comprised calcified atherosclerotic core plaques and matched proximal adjacent tissues from 3 carotid endarterectomy patients. Raw counts were processed using Seurat (v4.0): low-quality cells (<50 genes or >5% mitochondrial reads) were filtered, followed by log-normalization (scale factor = 10,000) and identification of 1500 highly variable genes (vst method). Dimensionality reduction was performed via PCA (top 20 PCs selected using JackStraw and elbow plots), and cells were clustered (Louvain algorithm, resolution = 0.5) before t-Distributed Stochastic Neighbor Embedding visualization. Marker genes for each cluster were identified (min.pct = 0.25, log_2_FC > 0.5) and annotated against the Human Primary Cell Atlas (SingleR) to define cell types. Finally, an independent validation dataset (GSE100927) was used for further confirmation of the target genes. Additionally, we employed the drug–gene interaction database (DGIdb) platform (DGIdb – Mining the druggable genome)^[23]^ to explore and identify potential drug candidates for the validated target genes based on their integrated scores, On the DGIdb platform, we selected candidate drugs based on their integrated scores. The integrated score is calculated based on multiple factors, including the strength of the drug–gene interaction evidence, the number of studies supporting the interaction, and the relevance of the drugs mechanism of action to the functions of the target genes. We focused on drugs with a high integrated score (above the 75th percentile of all available drugs for each gene) and those that have been experimentally validated in at least one in vitro or in vivo study related to the target genes function, aiming to uncover promising drug targets.

### 2.6. Construction and validation of the diagnostic model

We developed a comprehensive diagnostic model based on the identification of feature genes to predict the risk of PAS. Calibration curves were used to assess the agreement between the predicted probabilities of the model and the actual observed probabilities. A well-calibrated model would show a close fit between the predicted and observed values, with points lying close to the 45-degree line. Decision curve analysis (DCA) evaluated the net benefit of the model at different probability thresholds. It compared the benefits of using the model for decision-making (such as predicting PAS) against the risks of false positives and false negatives. A higher net benefit at a wide range of thresholds indicated a more useful model. Receiver operating characteristic (ROC) curves were generated by plotting the true positive rate against the false positive rate at different classification thresholds as previous studies.^[16,24,25]^ The area under the ROC curve (AUC) was used to quantify the models predictive ability. An AUC of 1 represents a perfect classifier, while an AUC of 0.5 indicates a model with no predictive value. In our study, the closer the AUC is to 1, the better the diagnostic nomogram model performs in distinguishing between PAS patients and healthy controls

### 2.7. Statistical analysis

In order to investigate the potential causal relationship between gene eQTLs levels and PAS, we conducted analyses using the Mendelian randomization software package (version 0.4.3). We employed the inverse-variance weighted (IVW) method,^[26]^ simple mode,^[27]^ weighted median,^[28]^ and MR Egger method.^[29,30]^ For the IVW method, we calculated the odds ratio (OR) to estimate the impact of exposure (eQTLs) on the outcome (PAS). The MR Egger regression was used to test for pleiotropy. We also performed a Cochrans *Q* test for heterogeneity analysis. Leave-one-out analysis was carried out to assess the influence of individual single nucleotide polymorphisms (SNPs) on the estimated results, further ensuring the reliability of our findings.^[31]^ Additionally, leave-one-out analysis was performed to evaluate the potential influence of SNPs on estimated results and further ensure the reliability of the findings.^[32]^ Data analysis involved the use of scatter plots, funnel plots, and forest plots. Scatter plots demonstrated the sensitivity of the results to outliers, funnel plots confirmed the robustness of the correlation and lack of heterogeneity, while forest plots illustrated the interaction between eQTL levels and the final instruments.

## 3. Results

### 3.1. Differential gene screening between normal and PAS tissues

**Figure 1.**
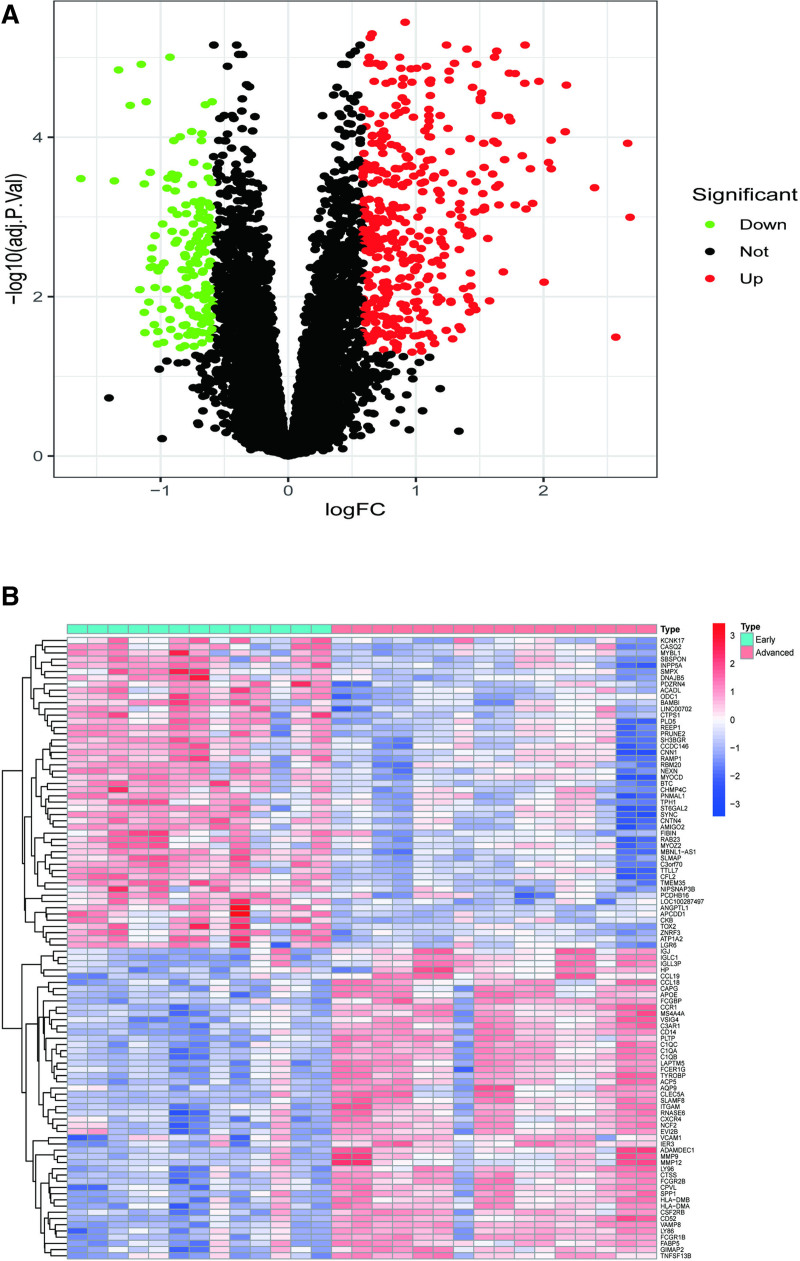
Comparative analysis of DEGs expression in PAS patients and healthy controls. (A) Volcano plot; (B) heatmap. In the volcano plot, red dots denote upregulated genes, green dots represent downregulated genes, and black dots indicate genes with no significant differential expression. In the heat map; red shows up-regulated genes and blue shows down-regulated genes. Advanced = experimental group, DEGs = differentially expressed genes, Early = control group, FC = folding change, PAS = peripheral atherosclerosis, PVal = adjusted *P* value.

Regarding the sample size, we conducted a power analysis prior to the study. Based on previous studies and the expected effect sizes of the DEGs, our sample sizes in GSE28829 and GSE100927 were estimated to have a power of over 80% to detect the observed gene expression differences with a significance level of *P* < .05. We have identified 571 DEGs (*P* < .05, |log_2_FC| ≥ 0.585. Visualization of these DEGs is depicted in heatmaps (Fig. 1A) and volcano plots (Fig. 1B).

### 3.2. Functional enrichment analysis using GO and KEGG pathway analysis

**Figure 2.**
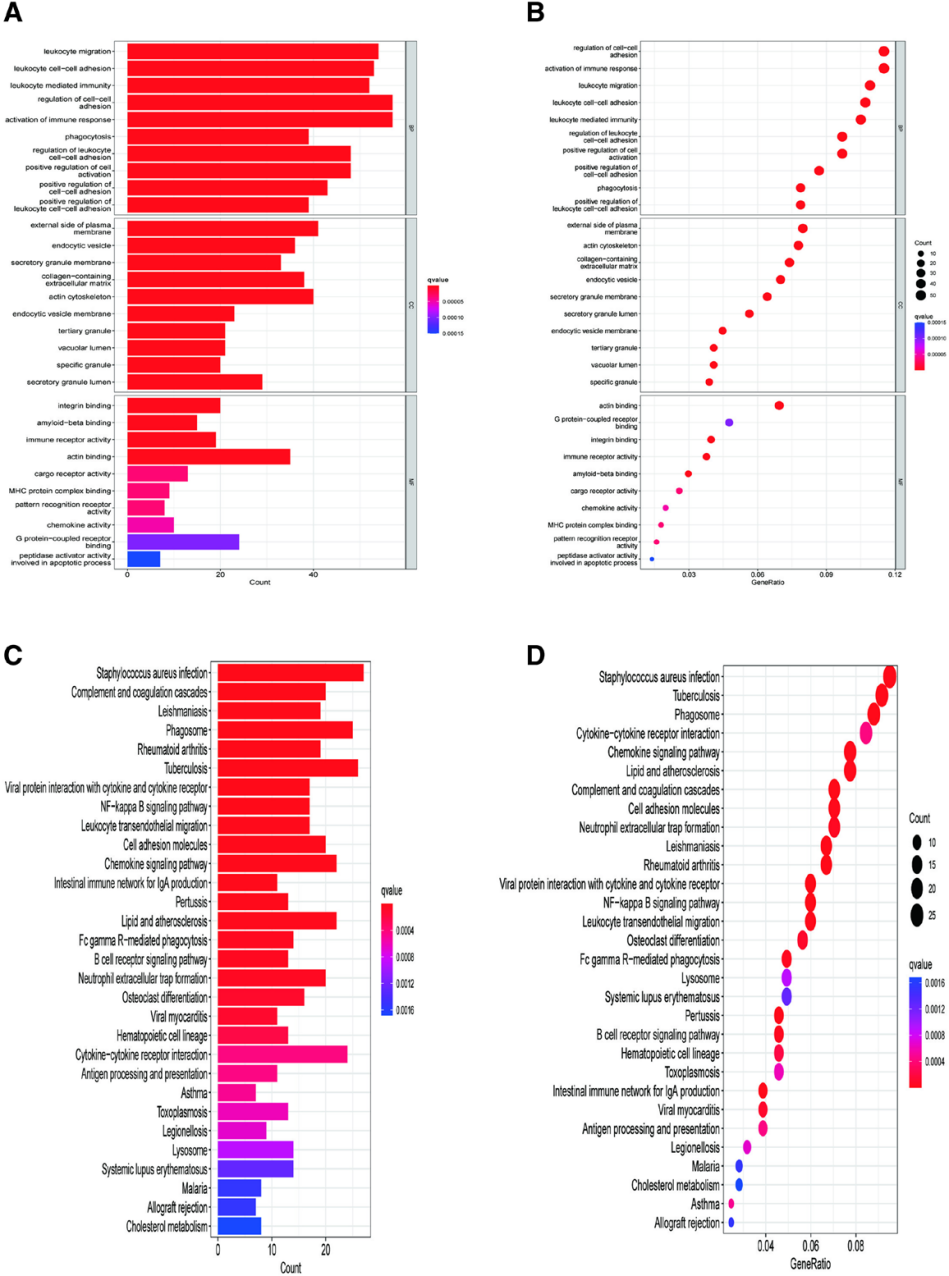
GO terms and KEGG pathway enrichment for differential gene analysis in peripheral artery disease. (A) Bar chart and (B) bubble chart for GO analysis. (C) Histogram and (D) bubble chart for KEGG analysis. In the bar chart, longer bars on the left indicate richer gene sets, and darker red colors signify higher significance. The bubble chart shows that the size of each bubble represents the number of enriched genes, with larger bubbles indicating greater gene enrichment. BP = biological process, CC = cellular component, GO = Gene Ontology, KEGG = Kyoto Encyclopedia of Genes and Genomes, MF = molecular function.

The functional roles of the identified DEGs were explored through GO and KEGG enrichment analyses (Fig. 2A–D). In the GO analysis (Fig. 2A, B), significantly enriched BP terms encomPASsed leukocyte migration, leukocyte cell–cell adhesion, and activation of immune response. Enriched CC terms included the external side of the plasma membrane and the actin cytoskeleton, while MF terms encomPASsed integrin binding, amyloid-beta binding, and actin binding. KEGG pathway analysis identified significantly enriched pathways such as *Staphylococcus aureus* infection, phagosome, tuberculosis, and the chemokine signaling pathway (Fig. 2C, D).

### 3.3. Selection of target genes

**Figure 3.**
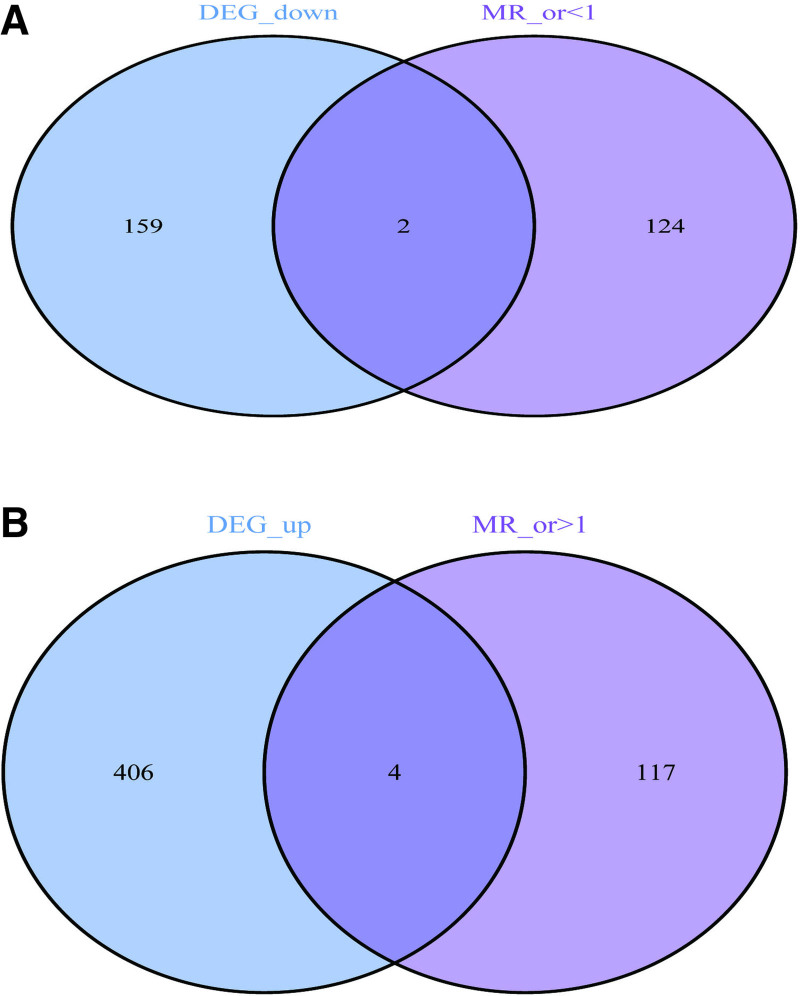
Venn diagram of differentially expressed genes and eQTLs in PAS. (A) Downregulated genes; (B) upregulated genes. eQTLs = expression quantitative trait loci, PAS = peripheral atherosclerosis.

**Figure 4.**
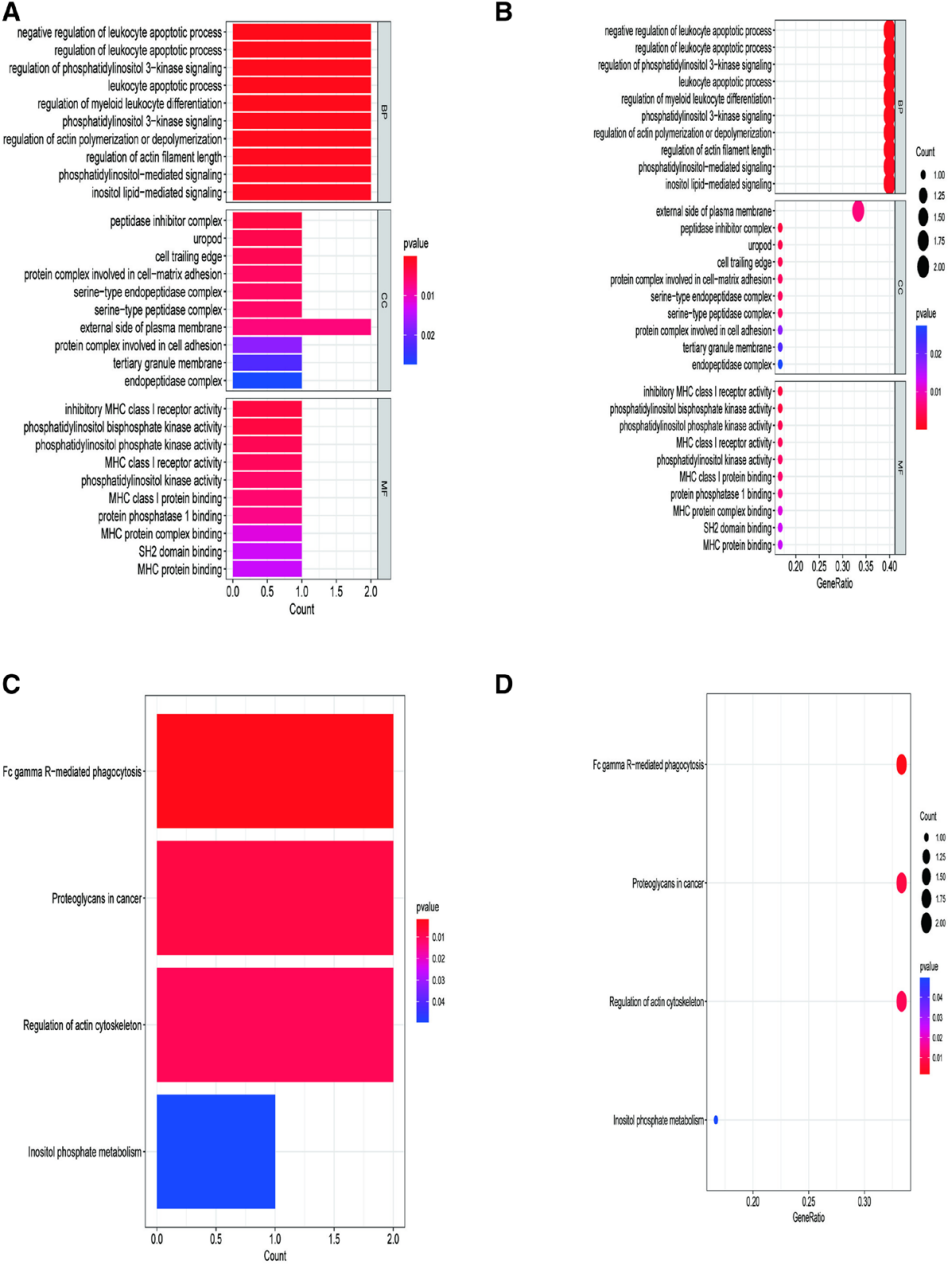
GO and KEGG analysis of target genes. (A) GO analysis bar chart; (B) GO analysis bubble chart; (C) KEGG analysis bar chart; (D) KEGG analysis bubble chart. GO = Gene Ontology, KEGG = Kyoto Encyclopedia of Genes and Genomes.

In the R environment, we employed the “VennDiagram” package to intersect the DEGs from the experimental group GSE28829 with the gene eQTLs data filtered based on standard criteria. A total of 6 genes were identified at the intersection, namely leukocyte immunoglobulin-like receptor B1 (LILRB1), hematopoietic cell-specific lyn substrate 1 (HCLS1), plasminogen activator urokinase (PLAU), C-type lectin domain family 2 member B (CLEC2B), phosphatidylinositol-4-phosphate 5-kinase type 1 beta (PIP5K1B), and cofilin 2 (CFL2). Among these, 4 genes, LILRB1, HCLS1, PLAU, and CLEC2B, exhibited upregulation (Fig. 3A), while 2 genes, PIP5K1B and CFL2, showed downregulation (Fig. 3B). The functional roles of the intersected genes were elucidated through GO and KEGG enrichment analyses within the R environment. In the GO analysis (Fig. 4A, B), significantly enriched BP terms included negative regulation of leukocyte apoptotic process and regulation of leukocyte apoptotic process. Enriched CC terms encomPASsed the peptidase inhibitor complex and uropod, while MF terms included inhibitory major histocompatibility complex class I receptor activity and phosphatidylinositol bisphosphate kinase activity. KEGG pathway analysis highlighted significantly enriched pathways such as Fc gamma R-mediated phagocytosis and Proteoglycans in cancer (Fig. 4C, D).

### 3.4. Causal relationship between eQTLs related genes and PAS

**Figure 5.**
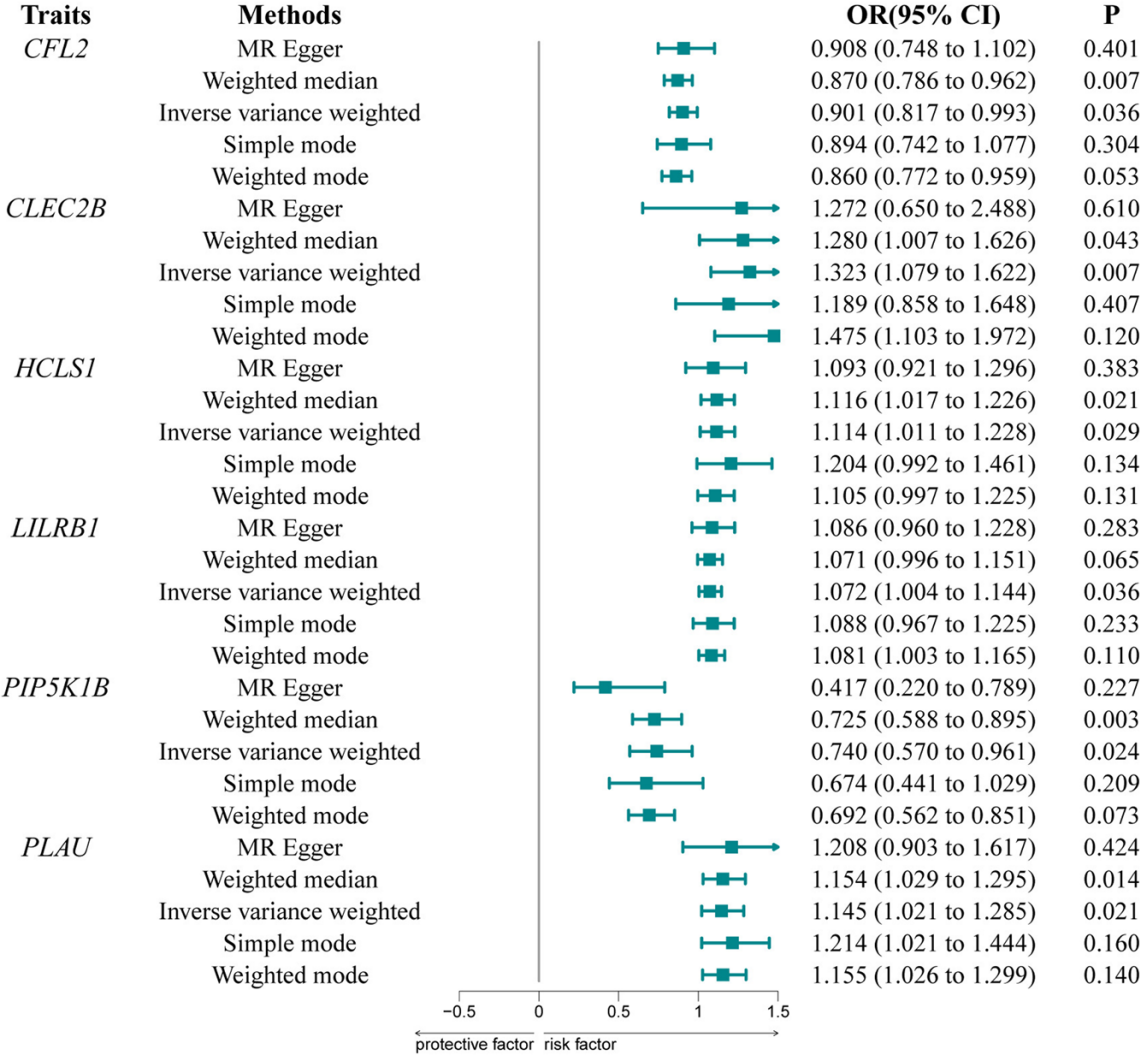
Forest plot depicting the causal relationship between target genes and PAS. IVW = inverse-variance weighted, CI = confidence interval, PAS = peripheral atherosclerosis.

To explore the causal association between eQTLs gene expression levels and PAS, we conducted MR analysis on 2 datasets, employing the IVW method as the primary analytical approach. Following instrumental variable selection and adjustment, all SNPs exhibited F statistics exceeding 10, indicative of robust instrumental variables. The outcomes of the IVW analysis (Fig. 5) indicated that CFL2 (OR = 0.901, 95% confidence interval [CI]: 0.817–0.993, *P* = .036) was associated with PAS, with PIP5K1B (OR = 0.740, 95% CI: 0.570–0.961, *P* = .024) identified as a protective factor against arteriosclerosis. Elevated expression levels of CFL2 and PIP5K1B genes correlated with reduced prevalence of PAS. Conversely, genes such as CLEC2B, HCLS1, and PLAU exhibited strong associations with PAS, with increased expression predicting higher PAS risk. Specifically, CLEC2B (OR = 1.323, 95% CI: 1.079–1.622, *P* = .007), HCLS1 (OR = 1.114, 95% CI: 1.011–1.228, *P* = .029), LILRB1 (OR = 1.072, 95% CI: 1.004–1.144, *P* = .036), and PLAU (OR = 1.145, 95% CI: 1.021–1.28, *P* = .021) were identified as risk factors for PAS. No evidence of pleiotropy or heterogeneity was observed based on the MR-Egger intercept test and Cochrans *Q* test (Tables S1 and S2, Supplemental Digital Content, https://links.lww.com/MD/O916).

### 3.5. Development of a nomogram model for predicting PAS based on feature genes

**Figure 6.**
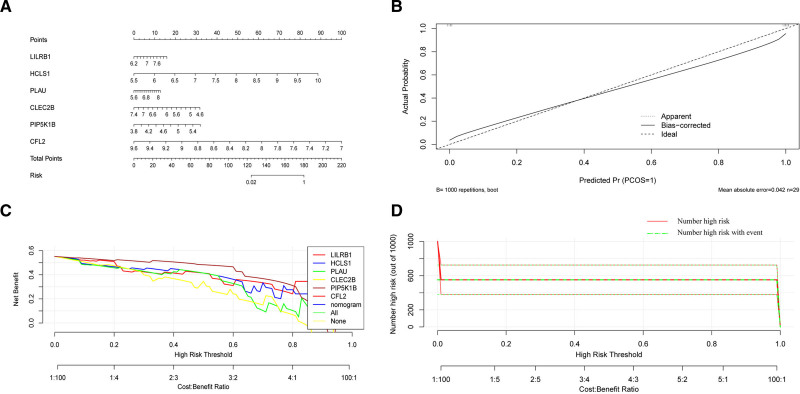
Construction and validation of the diagnostic model for PAS; (A) nomogram for PAS; (B) calibration curve of the model; (C) decision curve analysis; (D) clinical impact curve. PAS = peripheral atherosclerosis.

Based on the feature genes (LILRB1, HCLS1, PLAU, CLEC2B, PIP5K1B, and CFL2), we developed a diagnostic nomogram model for PAS. Using R software, we generated the diagnostic nomogram (Fig. 6A), calibration plot (Fig. 6B), DCA(Fig. 6C), and clinical impact curve (Fig. 6D). The results indicated that the calibration curve and DCA of the PAS diagnostic model demonstrated good fit. Across all practical risk thresholds (ranging from 0 to 1.0), the nomogram consistently exhibited excellent overall net benefit, significantly influencing patient prognosis. Furthermore, the clinical impact curve showed that the “number of high-risk patients” closely aligned with the “number of high-risk patients with events,” further underscoring the nomograms remarkable predictive capability for PAS. To use the nomogram, first locate each predictor value on its corresponding axis. Then, draw a vertical line from each value to the Points axis and sum all the individual points. Finally, draw a line from the Total Points axis to the Probability axis to obtain the predicted probability of PAS. A higher probability indicates a greater likelihood of PAS, while a lower probability corresponds to reduced risk. This model provides a user-friendly and intuitive tool for integrating multiple genetic markers into clinical decision-making.

### 3.6. Evaluation of the nomogram model using training and validation sets

**Figure 7.**
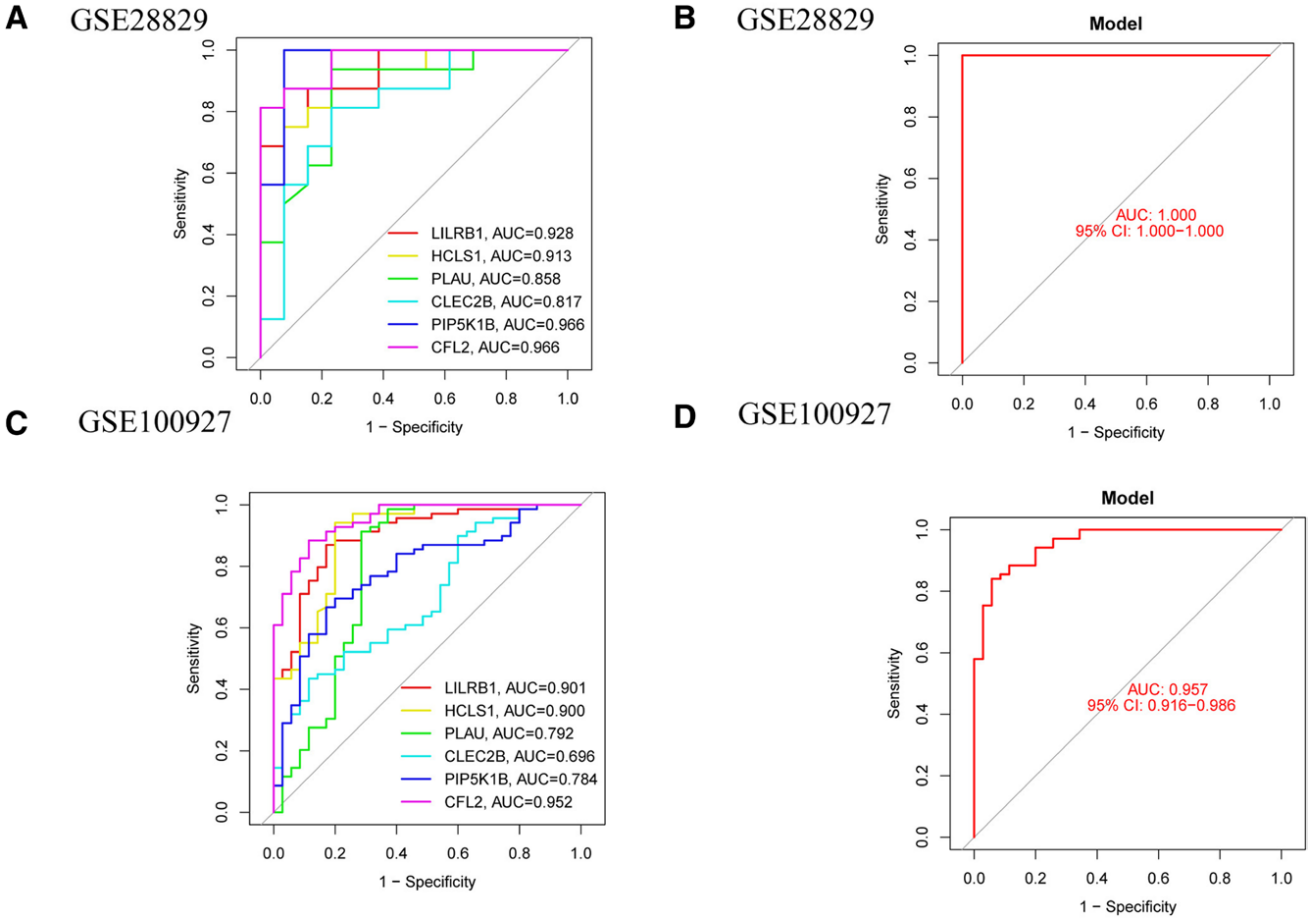
Validation of the nomogram model using ROC curves; (A) ROC and AUC for each common feature gene in GSE28829; (B) ROC and AUC for the nomogram model in GSE28829; (C) ROC and AUC for each common feature gene in GSE100927; (D) ROC and AUC for the nomogram model in GSE100927. AUC = area under the ROC curve, ROC = receiver operating characteristic.

In our study on the diagnostic efficacy of PAS, we utilized a nomogram model and evaluated its performance using 6 established common feature genes. The ROC curve analysis revealed that in the dataset GSE28829, the AUC values were as follows: LILRB1 at 0.928, HCLS1 at 0.913, PLAU at 0.858, CLEC2B at 0.817, and both PIP5K1B and CFL2 at 0.966 (Fig. 7A). Additionally, the nomogram model constructed from these 6 genes demonstrated exceptional accuracy, achieving an AUC of 1.0 with a 95% CI of 1.000 to 1.000 (Fig. 7B). In the validation dataset GSE100927, the AUC values were LILRB1 at 0.901, HCLS1 at 0.900, PLAU at 0.792, CLEC2B at 0.696, PIP5K1B at 0.784, and CFL2 at 0.952 (Fig. 7C). The nomogram model based on these 6 feature genes also exhibited high accuracy, with an AUC of 0.975 and a 95% CI of 0.916 to 0.986 (Fig. 7D). These results indicate that these 6 feature genes (LILRB1, HCLS1, PLAU, CLEC2B, PIP5K1B, and CFL2) and their combination model can serve as effective biomarkers for distinguishing PAS.

### 3.7. Validation of target genes

**Figure 8.**
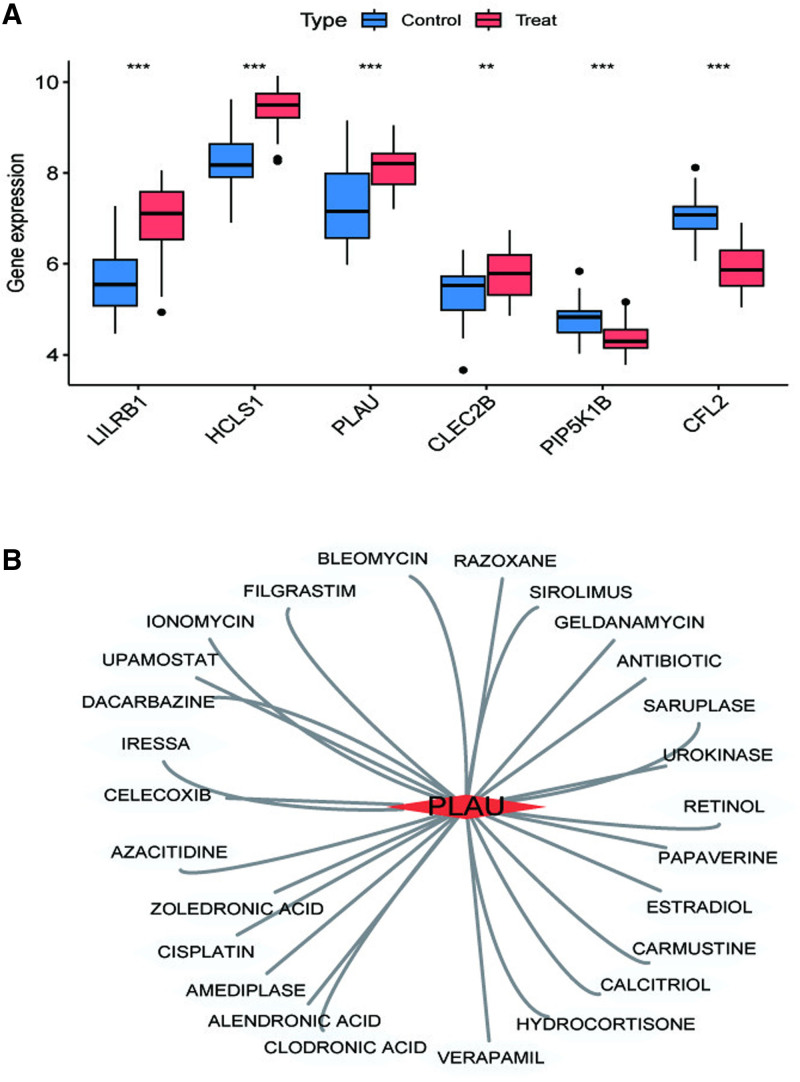
Validation and drug prediction of target genes. (A) Validation of target genes in the GSE100927 dataset; (B) drug prediction for target genes. ***P < .001, **P < .01, *P < .05. Control = normal individuals, Treat = patients with peripheral atherosclerosis.

**Figure 9.**
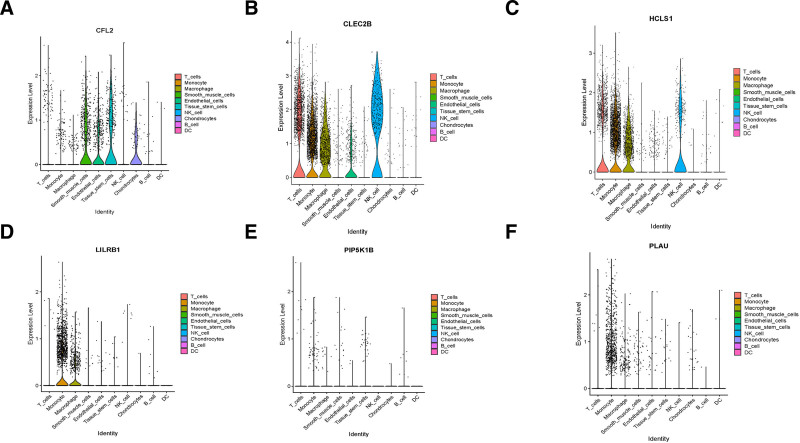
Visualization of the expression of target genes at the single-cell level.

The selected 6 key genes were validated in the validation dataset GSE100927 (Fig. 8A). Results revealed that, compared to normal tissues, 4 key genes were significantly upregulated, while 2 genes exhibited significant downregulation in peripheral arterial atherosclerosis. These findings closely mirrored those observed in the experimental dataset. Furthermore, leveraging the DGIdb website (DGIdb – Mining the Druggable Genome), candidate drug predictions were made based on the identified target genes. Ultimately, a total of 26 potential candidate drugs were discerned (Fig. 8B). To further evaluate the reliability of the 6 key genes, their expression levels were visualized across various cell types (Fig. 9). The analysis revealed that CFL2 is prominently expressed in smooth muscle cells and endothelial cells, while CLEC2B and HCLS1 demonstrate high expression levels in T cells, monocytes, and macrophages. Similarly, LILRB1 is predominantly expressed in monocytes and macrophages, whereas PIP5K1B and PLAU exhibit elevated expression levels in monocytes. Monocytes play a pivotal role in the pathogenesis and progression of PAS by mediating processes such as migration, differentiation, and inflammation. These cells act as a critical bridge linking systemic inflammatory responses to localized vascular damage. The functional involvement of monocytes aligns closely with the expression profiles of the identified key genes, providing valuable insights into the molecular mechanisms underlying PAS.

## 4. Discussion

In this study, we performed a comprehensive analysis of gene expression patterns in PAS, revealing key molecular mechanisms and potential therapeutic targets. We identified 571 DEGs between normal and PAS tissues, demonstrating significant disease-associated transcriptional changes. Functional enrichment analysis implicated critical BPs in PAS pathogenesis, including leukocyte migration, immune response activation, and cell adhesion, reinforcing the inflammatory nature of the disease. KEGG pathway analysis further highlighted enriched pathways such as Staphylococcus aureus infection, phagosome activity, and chemokine signaling, underscoring the central role of immune dysregulation in PAS progression. These findings align with established evidence linking chronic inflammation to atherosclerosis, with PAS emerging as a distinct clinical manifestation of this process.^[33,34]^ Kobiyama and Leys seminal work corroborates the critical role of dysregulated immune cell recruitment and inflammatory modulation in atherosclerosis progression, lending further credence to our findings.^[2]^ The identification of specific genes and pathways associated with immune response processes provides mechanistic insights into the pathophysiology of PAS, potentially informing the development of targeted therapeutic interventions aimed at modulating the immune response in affected individuals. Our MR analysis using the IVW method revealed significant associations, suggesting potential causal relationships between gene expression and PAS. Notably, CFL2 and PIP5K1B emerged as potential protective factors, with odds ratios (ORs) of 0.901 and 0.740, respectively, indicating that higher expression of these genes may reduce PAS risk or severity. Conversely, CLEC2B, HCLS1, LILRB1, and PLAU were identified as risk factors, where elevated expression correlated with increased disease susceptibility. These findings highlight a dual regulatory role of genes in PAS pathogenesis, with some conferring protection and others promoting risk. Leukocyte immunoglobulin-like receptor B1 (LILRB1), also known as ILT2, LIR-1, or CD85j, is an inhibitory receptor expressed across multiple leukocyte subsets. It binds HLA class I molecules and the UL18 glycoprotein of human cytomegalovirus (HCMV), playing a key role in modulating immune cell activation.^[35]^

In the PAS context, upregulation of LILRB1 may modulate immune responses through its interaction with HLA class I molecules. This molecular interplay could potentially impair normal immune surveillance within the arterial wall, contributing to dysregulated inflammatory processes and subsequent plaque development. LILRB1 expression correlates with the late-stage differentiation of pathogen-specific T lymphocytes. Moreover, a discernible association exists between the prevalence of LILRB1 + CD56 + T cells and carotid intima-media thickness.^[36,37]^ Hematopoietic cell-specific Lyn substrate 1 (HCLS1) orchestrates various biological phenomena encompassing intracellular signal transduction facilitation, protein phosphorylation enhancement, and transcriptional activity regulation entailing DNA templating. White blood cell recruitment to inflammatory sites hinges on dynamic actin remodeling, pivotal for their adhesion, migration, phagocytosis, and degranulation. Notably, HCLS1 exerts regulatory control over Arp 2/3-dependent migration within distinct leukocyte subsets. Persistent activation syndrome (PAS), a chronic inflammatory condition, potentially implicates the pathogenic influence of HCLS1.^[38,39]^ Upregulation of HCLS1 may enhance leukocyte recruitment and activation at atherosclerotic lesion sites by modulating actin cytoskeletal remodeling. This mechanism could facilitate immune cell infiltration, particularly monocytes and macrophages, thereby exacerbating local inflammatory responses. Plasminogen activator urokinase (PLAU) encodes a serine protease pivotal for plasminogen conversion to plasmin. PLAU exerts a significant influence on a myriad of biological and pathological processes including chemotaxis, cell adhesion, migration, proliferation, fibrinolysis, proteolysis, angiogenesis, inflammation, and neointima formation.^[40,41]^ Moreover, PLAU stands implicated in atherosclerotic plaque development and acute myocardial infarction. Studies on Apoe −/− mice underscore the deleterious effects of macrophage-specific PLAU overexpression, leading to accelerated atherosclerosis, coronary artery occlusion, and premature demise.^[42]^ Notably, a strong association between PLAU and acute myocardial infarction has been elucidated, particularly evidenced by the SNP rs4065 in the Chinese Han population.^[43]^ CLEC2B, belonging to the C-type lectin domain family 2, encodes members of the C-type lectin/C-type lectin-like domain (CTL/CTLD) superfamily. These members, characterized by a conserved protein fold, serve diverse functions spanning cell adhesion, intercellular signaling, glycoprotein turnover, and participation in inflammatory and immune responses. Noteworthy, sCLEC-2 emerges as a pivotal regulator of atherosclerotic thrombosis, whereas human CLEC-2, encoded by CLEC1B, functions as a non-classical C-type lectin receptor. Upon binding with splenic tyrosine kinase (Syk), CLEC-2 cytoplasmic tail dimerization enhances susceptibility to activation, aggregation, and thrombosis.^[44]^ Additionally, CLEC-2 assumes a crucial role in phagocytosis, pivotal for macrophage infiltration and foam cell formation during atherogenesis.^[45]^ PIP5K1B is implicated in 1-phosphatidylinositol-4-phosphate 5-kinase activity and regulation of phosphatidylinositol 3-kinase signaling, along with upstream control of phosphatidylinositol biosynthesis. PIP5K1B may promote cell proliferation in certain cancer-related contexts, which appears to contrast with its potential protective role in PAS. These discrepancies could stem from differences in study populations, experimental approaches, or the complex regulatory mechanisms of PIP5K1B across tissues and disease states. Further research is needed to clarify these differences and fully elucidate the role of PIP5K1B in PAS.^[46]^ Cofilin 2 (CFL2), encoding an intracellular protein, critically regulates actin-filament dynamics. CFL2 serves as a prominent constituent of both intranuclear and cytoplasmic actin rods, exerting reversible control over actin polymerization and depolymerization in a pH-dependent manner, via binding G- and F-actin in a 1:1 ratio. Notably, mutations within CFL2 precipitate nemaline myopathy type 7, a specific congenital myopathy.^[47]^ Matrix metallopeptidase 9, also acknowledged as matrix metallopeptidase 9, assumes a pivotal role in atherosclerosis pathogenesis.^[48]^ Analogously, cystic fibrosis transmembrane conductance regulator shares functional parallels with ATP-binding cassette subfamily A member 1. Research by Schmitz,^[49]^ underscores ATP-binding cassette subfamily A member 1s prominent role in modulating plasma high-density lipoprotein cholesterol, pivotal in impeding atherosclerosis progression. Moreover, Wang et al have identified CFL2 as an interacting partner of cystic fibrosis transmembrane conductance regulator.^[50,51]^ The diagnostic nomogram model based on 6 feature genes demonstrated excellent predictive performance. In the training cohort (GSE28829), it achieved an AUC of 1.0, indicating perfect discrimination between PAS patients and healthy controls. In the validation cohort (GSE100927), the model maintained high accuracy, with an AUC of 0.975. At a clinically relevant probability threshold (e.g., 0.5), the model achieved a sensitivity of approximately 90% and a specificity of around 95% in the validation set. This indicates that the model correctly identified 90% of PAS patients (true positives) and accurately excluded 95% of healthy controls (true negatives). Compared with other diagnostic or risk-scoring models for PAS reported in the literature, the diagnostic nomogram model of this study exhibits significant advantages. Traditional models predominantly rely on a limited number of indicators, whereas our model integrates multiple key genes, offering a more comprehensive assessment of disease risk. In terms of accuracy, the high AUC values of our model in both the training and validation sets outperform those of some models reported in the literature.

However, the performance of the nomogram model may be influenced by factors such as population heterogeneity and the presence of comorbidities. Therefore, further validation in larger and more diverse populations is essential to fully assess its clinical utility. Open data has its internal limitations.^[52,53]^ In addition, by leveraging the DGIdb database, we identified 26 potential candidate drugs targeting these 6 genes, thereby enhancing the translational potential of our genomic findings. If validated for efficacy in future studies, these agents may offer novel therapeutic strategies for the treatment or management of PAS, potentially improving outcomes for patients afflicted by this debilitating condition. Despite these promising findings, our study has several limitations. One notable limitation is the reliance on gene expression data derived from tissue samples. Tissue-based gene expression may not accurately capture the dynamic changes occurring in circulating immune cells such as monocytes and lymphocytes, which play crucial roles in the pathogenesis of PAS. For instance, monocytes recruited to atherosclerotic lesions may exhibit altered gene expression profiles that are not reflected in static tissue samples. Moreover, tissue gene expression is influenced by the local microenvironment, which may not accurately represent the systemic disease state. Another limitation concerns the generalizability of our findings, as the study population was predominantly of European ancestry. Additionally, while the nomogram model demonstrated high AUC values, it necessarily simplifies the complex, multifactorial nature of PAS. Unaccounted factors – such as epigenetic modifications, protein–protein interactions, and environmental influences – may significantly impact disease progression and therapeutic response. Although MR analyses were conducted to mitigate pleiotropy and heterogeneity, residual confounding – such as horizontal pleiotropy or weak instrument bias – cannot be fully excluded, potentially affecting causal inference. Future investigations should employ prospective, multicenter, and multi-ethnic cohort studies incorporating multi-omics data to validate the identified molecular associations. Systematic functional validation of key target genes should be conducted, utilizing in vitro cellular models to elucidate their molecular mechanisms and employing genetically engineered animal models to delineate their pathophysiological roles in PAS progression. Concurrently, translational research should be advanced through standardized preclinical pharmacodynamic evaluations and well-designed clinical trials to comprehensively assess the efficacy and safety profiles of candidate therapeutic agents. Furthermore, the clinical utility of diagnostic models requires rigorous validation across diverse healthcare settings (e.g., high-risk population screening), with particular emphasis on exploring their potential applications in personalized medicine, thereby facilitating the development of novel precision medicine strategies for PAS management.

## 5. Conclusion

This study identified 6 feature genes linked to PAS and validated their diagnostic and therapeutic potential. The developed diagnostic model exhibited strong predictive performance, while the identified candidate drugs offer promising avenues for targeted PAS treatment. These findings enhance our understanding of PAS molecular mechanisms and support improved clinical management strategies.

## Acknowledgments

I sincerely appreciate the shared public database resources, which have been invaluable to my research and enhanced the quality of this study.

## Author contributions

**Conceptualization:** Luofei Huang.

**Data curation:** Luofei Huang, Quanzhi Lin.

**Formal analysis:** Luofei Huang, Han Li, Quanzhi Lin.

**Funding acquisition:** Quanzhi Lin.

**Investigation:** Quanzhi Lin.

## Supplementary Material



## References

[1] Libby P Buring JE Badimon L. Atherosclerosis. Nat Rev Dis Primers. 2019;5:56. 31420554 10.1038/s41572-019-0106-z

[2] Kobiyama K Ley K. Atherosclerosis. Circ Res. 2018;123:1118–20. 30359201 10.1161/CIRCRESAHA.118.313816 PMC6298754

[3] Herrington W Lacey B Sherliker P Armitage J Lewington S. Epidemiology of atherosclerosis and the potential to reduce the global burden of atherothrombotic disease. Circ Res. 2016;118:535–46. 26892956 10.1161/CIRCRESAHA.115.307611

[4] Hetherington I Totary-Jain H. Anti-atherosclerotic therapies: milestones, challenges, and emerging innovations. Mol Ther. 2022;30:3106–17. 36065464 10.1016/j.ymthe.2022.08.024 PMC9552812

[5] Majithia A Bhatt DL. Novel antiplatelet therapies for atherothrombotic diseases. Arterioscler Thromb Vasc Biol. 2019;39:546–57. 30760019 10.1161/ATVBAHA.118.310955 PMC6445601

[6] Liu Y Chen KJ. Atherosclerosis, vascular aging and therapeutic strategies. Chin J Integr Med. 2012;18:83–7. 22311404 10.1007/s11655-012-0996-z

[7] Hedin U Matic LP. Recent advances in therapeutic targeting of inflammation in atherosclerosis. J Vasc Surg. 2019;69:944–51. 30591299 10.1016/j.jvs.2018.10.051

[8] Lusis AJ Mar R Pajukanta P. Genetics of atherosclerosis. Annu Rev Genomics Hum Genet. 2004;5:189–218. 15485348 10.1146/annurev.genom.5.061903.175930

[9] Zhu Z Zhang F Hu H. Integration of summary data from GWAS and eQTL studies predicts complex trait gene targets. Nat Genet. 2016;48:481–7. 27019110 10.1038/ng.3538

[10] Wang SS Schadt EE Wang H. Identification of pathways for atherosclerosis in mice: integration of quantitative trait locus analysis and global gene expression data. Circ Res. 2007;101:e11–30. 17641228 10.1161/CIRCRESAHA.107.152975

[11] Gupta V Walia GK Sachdeva MP. “Mendelian randomization”: an approach for exploring causal relations in epidemiology. Public Health. 2017;145:113–9. 28359378 10.1016/j.puhe.2016.12.033

[12] Sekula P Del Greco M F Pattaro C Köttgen A. Mendelian randomization as an approach to assess causality using observational data. J Am Soc Nephrol. 2016;27:3253–65. 27486138 10.1681/ASN.2016010098 PMC5084898

[13] Bowden J Holmes MV. Meta-analysis and mendelian randomization: a review. Res Synth Methods. 2019;10:486–96. 30861319 10.1002/jrsm.1346 PMC6973275

[14] Birney E. Mendelian Randomization. Cold Spring Harb Perspect Med. 2022;12:a041302. 34872952 10.1101/cshperspect.a041302 PMC9121891

[15] Barrett T Wilhite SE Ledoux P. NCBI GEO: archive for functional genomics data sets--update. Nucleic Acids Res. 2013;41:D991–5. 23193258 10.1093/nar/gks1193 PMC3531084

[16] Liu H Weng J. A comprehensive bioinformatic analysis of cyclin-dependent kinase 2 (CDK2) in glioma. Gene. 6325;822:146325. 35183683 10.1016/j.gene.2022.146325

[17] Liu H Tang T. A bioinformatic study of IGFBPs in glioma regarding their diagnostic, prognostic, and therapeutic prediction value. Am J Transl Res. 2023;15:2140–55. 37056850 PMC10086936

[18] Liu H. Association between sleep duration and depression: a Mendelian randomization analysis. J Affect Disord. 2023;335:152–4. 37178827 10.1016/j.jad.2023.05.020

[19] Liu H Xie R Dai Q Fang J Xu Y Li B. Exploring the mechanism underlying hyperuricemia using comprehensive research on multi-omics. Sci Rep. 2023;13:7161. 37138053 10.1038/s41598-023-34426-y PMC10156710

[20] Bowden J Spiller W Del Greco M F. Improving the visualization, interpretation and analysis of two-sample summary data Mendelian randomization via the Radial plot and Radial regression. Int J Epidemiol. 2018;47:1264–78. 29961852 10.1093/ije/dyy101 PMC6124632

[21] Elhage KG Kranyak A Jin JQ. Mendelian randomization studies in atopic dermatitis: a systematic review. J Invest Dermatol. 2024;144:1022–37. 37977498 10.1016/j.jid.2023.10.016

[22] Vaucher J Keating BJ Lasserre AM. Cannabis use and risk of schizophrenia: a Mendelian randomization study. Mol Psychiatry. 2018;23:1287–92. 28115737 10.1038/mp.2016.252 PMC5984096

[23] Wagner AH Coffman AC Ainscough BJ. DGIdb 2.0: mining clinically relevant drug–gene interactions. Nucleic Acids Res. 2016;44:D1036–44. 26531824 10.1093/nar/gkv1165 PMC4702839

[24] Liu H Weng J. A pan-cancer bioinformatic analysis of RAD51 regarding the values for diagnosis, prognosis, and therapeutic prediction. Front Oncol. 2022;12:858756. 35359409 10.3389/fonc.2022.858756 PMC8960930

[25] Liu H Weng J Huang CL-H Jackson AP. Is the voltage-gated sodium channel β3 subunit (SCN3B) a biomarker for glioma? Funct Integr Genomics. 2024;24:162. 39289188 10.1007/s10142-024-01443-7

[26] Burgess S Small DS Thompson SG. A review of instrumental variable estimators for Mendelian randomization. Stat Methods Med Res. 2017;26:2333–55. 26282889 10.1177/0962280215597579 PMC5642006

[27] Dai Z Xu W Ding R. Two-sample Mendelian randomization analysis evaluates causal associations between inflammatory bowel disease and osteoporosis. Front Public Health. 2023;11:1151837. 37304119 10.3389/fpubh.2023.1151837 PMC10250718

[28] Bowden J Davey Smith G Haycock PC Burgess S. Consistent estimation in mendelian randomization with some invalid instruments using a weighted median estimator. Genet Epidemiol. 2016;40:304–14. 27061298 10.1002/gepi.21965 PMC4849733

[29] Bowden J Del Greco M F Minelli C Davey Smith G Sheehan NA Thompson JR. Assessing the suitability of summary data for two-sample Mendelian randomization analyses using MR-Egger regression: the role of the I2 statistic. Int J Epidemiol. 2016;45:1961–74. 27616674 10.1093/ije/dyw220 PMC5446088

[30] Burgess S Thompson SG. Interpreting findings from Mendelian randomization using the MR-Egger method. Eur J Epidemiol. 2017;32:377–89. 28527048 10.1007/s10654-017-0255-x PMC5506233

[31] Verbanck M Chen C-Y Neale B Do R. Detection of widespread horizontal pleiotropy in causal relationships inferred from Mendelian randomization between complex traits and diseases. Nat Genet. 2018;50:693–8. 29686387 10.1038/s41588-018-0099-7 PMC6083837

[32] Wei D Jiang Y Cheng J Wang H Sha K Zhao J. Assessing the association of leukocyte telomere length with ankylosing spondylitis and rheumatoid arthritis: a bidirectional Mendelian randomization study. Front Immunol. 2023;14:1023991. 37033949 10.3389/fimmu.2023.1023991 PMC10080099

[33] Whicher J Biasucci L Rifai N. Inflammation, the acute phase response and atherosclerosis. Clin Chem Lab Med. 1999;37:495–503. 10418738 10.1515/CCLM.1999.080

[34] Aday AW Matsushita K. Epidemiology of peripheral artery disease and polyvascular disease. Circ Res. 2021;128:1818–32. 34110907 10.1161/CIRCRESAHA.121.318535 PMC8202714

[35] Cosman D Fanger N Borges L. A novel immunoglobulin superfamily receptor for cellular and viral MHC class I molecules. Immunity. 1997;7:273–82. 9285411 10.1016/s1074-7613(00)80529-4

[36] Antrobus RD Khan N Hislop AD. Virus-specific cytotoxic T lymphocytes differentially express cell-surface leukocyte immunoglobulin-like receptor-1, an inhibitory receptor for class I major histocompatibility complex molecules. J Infect Dis. 2005;191:1842–53. 15871117 10.1086/429927

[37] Romo N Fitó M Gumá M. Association of atherosclerosis with expression of the LILRB1 receptor by human NK and T-cells supports the infectious burden hypothesis. Arterioscler Thromb Vasc Biol. 2011;31:2314–21. 21817101 10.1161/ATVBAHA.111.233288

[38] Castro-Ochoa KF Guerrero-Fonseca IM Schnoor M. Hematopoietic cell-specific lyn substrate (HCLS1 or HS1): a versatile actin-binding protein in leukocytes. J Leukoc Biol. 2019;105:881–90. 30537294 10.1002/JLB.MR0618-212R

[39] Skokowa J Klimiankou M Klimenkova O. Interactions among HCLS1, HAX1 and LEF-1 proteins are essential for G-CSF-triggered granulopoiesis. Nat Med. 2012;18:1550–9. 23001182 10.1038/nm.2958 PMC3941918

[40] Chavakis T Kanse SM May AE Preissner KT. Haemostatic factors occupy new territory: the role of the urokinase receptor system and kininogen in inflammation. Biochem Soc Trans. 2002;30:168–73. 12023845 10.1042/

[41] Song C Qiao Z Chen L. Identification of key genes as early warning signals of acute myocardial infarction based on weighted gene correlation network analysis and dynamic network biomarker algorithm. Front Immunol. 2022;13:879657. 35795669 10.3389/fimmu.2022.879657 PMC9251518

[42] Cozen AE Moriwaki H Kremen M. Macrophage-targeted overexpression of urokinase causes accelerated atherosclerosis, coronary artery occlusions, and premature death. Circulation. 2004;109:2129–35. 15096455 10.1161/01.CIR.0000127369.24127.03

[43] Xu J Li W Bao X. Association of putative functional variants in the PLAU gene and the PLAUR gene with myocardial infarction. Clin Sci (Lond). 2010;119:353–9. 20518747 10.1042/CS20100151

[44] Hughes CE Sinha U Pandey A Eble JA O'Callaghan CA Watson SP. Critical Role for an acidic amino acid region in platelet signaling by the HemITAM (hemi-immunoreceptor tyrosine-based activation motif) containing receptor CLEC-2 (C-type lectin receptor-2). J Biol Chem. 2013;288:5127–35. 23264619 10.1074/jbc.M112.411462 PMC3576117

[45] Kerrigan AM Dennehy KM Mourão-Sá D. CLEC-2 is a phagocytic activation receptor expressed on murine peripheral blood neutrophils. J Immunol. 2009;182:4150–7. 19299712 10.4049/jimmunol.0802808 PMC2727695

[46] van den Bout I Jones DR Shah ZH. Collaboration of AMPK and PKC to induce phosphorylation of Ser413 on PIP5K1B resulting in decreased kinase activity and reduced PtdIns(4,5)P2 synthesis in response to oxidative stress and energy restriction. Biochem J. 2013;455:347–58. 23909401 10.1042/BJ20130259

[47] Kondo E Nishimura T Kosho T. Recessive RYR1 mutations in a patient with severe congenital nemaline myopathy with ophthalomoplegia identified through massively parallel sequencing. Am J Med Genet A. 2012;158A:772–8. 22407809 10.1002/ajmg.a.35243

[48] Blin J Ahmad Z Rampal LRSOG Mohtarrudin N Tajudin AKH Adnan RS. Preliminary assessment of differential expression of candidate genes associated with atherosclerosis. Genes Genet Syst. 2013;88:199–209. 24025248 10.1266/ggs.88.199

[49] Schmitz G Buechler C. ABCA1: regulation, trafficking and association with heteromeric proteins. Ann Med. 2002;34:334–47. 12452478 10.1080/078538902320772098

[50] Wang X Venable J LaPointe P. Hsp90 cochaperone Aha1 downregulation rescues misfolding of CFTR in cystic fibrosis. Cell. 2006;127:803–15. 17110338 10.1016/j.cell.2006.09.043

[51] Lin M Zhao L Zhao W Weng J. Dissecting the mechanism of carotid atherosclerosis from the perspective of regulation. Int J Mol Med. 2014;34:1458–66. 25318463 10.3892/ijmm.2014.1960 PMC4214333

[52] Liu H Guo Z Wang P. Genetic expression in cancer research: challenges and complexity. Gene Rep. 2042;37:102042.

[53] Liu H Li Y Karsidag M Tu T Wang P. Technical and biological biases in bulk transcriptomic data mining for cancer research. J Cancer. 2025;16:34–43. 39744578 10.7150/jca.100922 PMC11660120

