# Supplementary material for: Identification of key genes and diagnostic biomarkers for peripheral atherosclerosis: A multi-omics approach
Source: Medicine (Baltimore). 2025 May 23;104(21):e42437. doi: 10.1097/MD.0000000000042437 (PMC12114065; doi:10.1097/MD.0000000000042437)
Supplement: Supplementary file 1 [file medi-104-e42437-s001.docx]

Supplementary Table 1.Analysis of Pleiotropy in Target Genes Associated with PAS;PAS,Peripheral Atherosclerosis.

| id.exposure | id.outcome | outcome | exposure | egger_intercept | se | pval |
| --- | --- | --- | --- | --- | --- | --- |
| eqtl-a-ENSG00000165410 | 13843J | Peripheral atherosclerosis | CFL2 | -0.00336 | 0.035314 | 0.930226 |
| eqtl-a-ENSG00000110852 | NhGPNw | Peripheral atherosclerosis | CLEC2B | 0.006843 | 0.056343 | 0.923059 |
| eqtl-a-ENSG00000180353 | wnrh16 | Peripheral atherosclerosis | HCLS1 | 0.00556 | 0.018985 | 0.788707 |
| eqtl-a-ENSG00000104972 | K5livr | Peripheral atherosclerosis | LILRB1 | -0.00753 | 0.030972 | 0.82359 |
| eqtl-a-ENSG00000107242 | gPVBmy | Peripheral atherosclerosis | PIP5K1B | 0.115836 | 0.062591 | 0.315378 |
| eqtl-a-ENSG00000122861 | K21Ssu | Peripheral atherosclerosis | PLAU | -0.01477 | 0.035059 | 0.746193 |

Supplementary Table 2.Heterogeneity Analysis of Target Genes in PAS;PAS,Peripheral Atherosclerosis.

| id.exposure | id.outcome | outcome | exposure | method | Q | Q_df | Q_pval |
| --- | --- | --- | --- | --- | --- | --- | --- |
| eqtl-a-ENSG00000165410 | 13843J | Peripheral atherosclerosis | CFL2 | MR Egger | 4.888613 | 3 | 0.180137 |
| eqtl-a-ENSG00000165410 | 13843J | Peripheral atherosclerosis | CFL2 | Inverse variance weighted | 4.903353 | 4 | 0.297359 |
| eqtl-a-ENSG00000110852 | NhGPNw | Peripheral atherosclerosis | CLEC2B | MR Egger | 1.225144 | 1 | 0.268353 |
| eqtl-a-ENSG00000110852 | NhGPNw | Peripheral atherosclerosis | CLEC2B | Inverse variance weighted | 1.243215 | 2 | 0.53708 |
| eqtl-a-ENSG00000180353 | wnrh16 | Peripheral atherosclerosis | HCLS1 | MR Egger | 4.392758 | 3 | 0.222058 |
| eqtl-a-ENSG00000180353 | wnrh16 | Peripheral atherosclerosis | HCLS1 | Inverse variance weighted | 4.518355 | 4 | 0.340377 |
| eqtl-a-ENSG00000104972 | K5livr | Peripheral atherosclerosis | LILRB1 | MR Egger | 3.731352 | 3 | 0.291973 |
| eqtl-a-ENSG00000104972 | K5livr | Peripheral atherosclerosis | LILRB1 | Inverse variance weighted | 3.804867 | 4 | 0.433058 |
| eqtl-a-ENSG00000107242 | gPVBmy | Peripheral atherosclerosis | PIP5K1B | MR Egger | 0.18173 | 1 | 0.669891 |
| eqtl-a-ENSG00000107242 | gPVBmy | Peripheral atherosclerosis | PIP5K1B | Inverse variance weighted | 3.606808 | 2 | 0.164737 |
| eqtl-a-ENSG00000122861 | K21Ssu | Peripheral atherosclerosis | PLAU | MR Egger | 1.828529 | 1 | 0.176301 |
| eqtl-a-ENSG00000122861 | K21Ssu | Peripheral atherosclerosis | PLAU | Inverse variance weighted | 2.152985 | 2 | 0.340789 |
